# Post‐progression survival after atezolizumab plus carboplatin and etoposide as first‐line chemotherapy in small cell lung cancer has a significant impact on overall survival

**DOI:** 10.1111/1759-7714.14621

**Published:** 2022-09-05

**Authors:** Ken Masubuchi, Hisao Imai, Satoshi Wasamoto, Takeshi Tsuda, Hiroyuki Minemura, Yoshiaki Nagai, Yutaka Yamada, Takayuki Kishikawa, Yukihiro Umeda, Ayako Shiono, Hiroki Takechi, Jun Shiihara, Kyoichi Kaira, Kenya Kanazawa, Hirokazu Taniguchi, Takayuki Kaburagi, Hiroshi Kagamu, Koichi Minato

**Affiliations:** ^1^ Division of Respiratory Medicine, Gunma Prefectural Cancer Center Ota Japan; ^2^ Department of Respiratory Medicine, Comprehensive Cancer Center, International Medical Center Saitama Medical University Hidaka Japan; ^3^ Division of Respiratory Medicine, Saku Central Hospital Advanced Care Center Saku Japan; ^4^ Division of Respiratory Medicine, Toyama Prefectural Central Hospital Toyama Japan; ^5^ Department of Pulmonary Medicine Fukushima Medical University Fukushima Japan; ^6^ Department of Respiratory Medicine Jichi Medical University, Saitama Medical Center Saitama Japan; ^7^ Division of Respiratory Medicine, Ibaraki Prefectural Central Hospital Kasama Japan; ^8^ Division of Thoracic Oncology, Tochigi Cancer Center Utsunomiya Japan; ^9^ Third Department of Internal Medicine, Faculty of Medical Sciences University of Fukui Eiheiji Japan

**Keywords:** atezolizumab, carboplatin, etoposide, overall survival, post‐progression survival

## Abstract

**Background:**

The effect of first‐line chemotherapy on overall survival (OS) may be significantly influenced by subsequent therapy for patients with extensive disease small cell lung cancer (ED‐SCLC). Therefore, we evaluated the relationship between progression‐free survival (PFS), post‐progression survival (PPS), and OS of ED‐SCLC patients treated with atezolizumab plus carboplatin and etoposide as first‐line therapy.

**Methods:**

We analyzed the data of 57 patients with relapsed ED‐SCLC treated with atezolizumab plus carboplatin and etoposide (AteCE) as first‐line chemotherapy between August 2019 and September 2020. The respective correlations between PFS‐OS and PPS‐OS following first‐line AteCE treatment were examined at the individual patient level.

**Results:**

Spearman's rank correlation analysis and linear regression analysis showed that PPS strongly correlated with OS (*r* = 0.93, *p* < 0.05, *R* ^*2*^ = 0.85) and that PFS moderately correlated with OS (*r* = 0.55, *p* < 0.05, *R* ^*2*^ = 0.28). Performance status at relapse (0–1/≥2), number of cycles of atezolizumab maintenance therapy (<3/≥3), and platinum rechallenge chemotherapy all significantly positively correlated with PPS (*p* < 0.05).

**Conclusions:**

Upon comparing OS‐PFS and OS‐PPS in this patient population, OS and PPS were found to have a stronger correlation. These results suggest that performance status at relapse, atezolizumab maintenance, or chemotherapy rechallenge could affect PPS.

## Graphical abstract

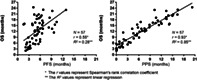

## INTRODUCTION

Lung cancer is the most common cause of cancer death worldwide.^1^ Small cell lung cancer (SCLC) is characterized by exponentially progressive disease and distant metastasis and accounts for 10%–15% of all lung malignancies.^2^ Approximately 70% of SCLC cases will have already reached the extensive disease (ED) stage, a stage related to poor prognosis, at initial diagnosis.^3^ Cytotoxic drug treatment can palliate and improve short‐term survival of most patients with ED‐SCLC, but long‐term survival is poor.^4^, ^5^ Until just a few years ago, when immune checkpoint inhibitors (ICIs) were introduced into the treatment of SCLC, one of the standard first‐line treatments for patients with ED‐SCLC was combination chemotherapy with platinum and etoposide. The median survival duration with platinum and etoposide combination chemotherapy was approximately 10 months, and no significant overall survival (OS) extension has been demonstrated for more than two decades.^6^, ^7^ ED‐SCLC is a malignant disease with a documented objective response rate (ORR) for first‐line treatment of 44%–78%, median progression‐free survival (PFS) of 4.3–5.7 months, median OS of 7.5–10.9 months, and 5‐year survival rate of only 2.8%.^7^, ^8^ As shown in the results of the IMpower133 and CASPIAN studies, since the recent adoption of ICIs, the survival of patients with ED‐SCLC has improved.^9^, ^10^ Thus, we evaluated patient outcomes for those who received atezolizumab plus carboplatin and etoposide (AteCE) as first‐line therapy because it is now regarded as one of the standard treatment choices for patients with ED‐SCLC. OS is typically short, and treatment strategies are scarce for ED‐SCLC patients.

Both PFS and OS are widely used endpoints in oncology clinical trials to assess survival; OS is a reliable, accurate measure and has the advantage of being easily calculated by describing the date of death. The influence of front‐line therapy on OS might be influenced by various treatment strategies.^11^ Conversely, PFS is easier to assess earlier than OS because its components are chronologically before those of OS.^12^ If there is a strong, significant relationship between PFS and OS, PFS may be an alternative indicator for OS. In non‐small‐cell lung cancer (NSCLC), prolonged PFS does not necessarily translate to prolonged OS,^13^ but post‐progression survival (PPS) is highly correlated with OS beyond first‐line treatment.^14^, ^15^ Several studies with individual‐level analysis have reported that PPS after first‐line treatment is strongly correlated with OS in metastatic NSCLC.^16^, ^17^, ^18^ Moreover, OS is represented by the summation of PFS and PPS.^11^ A strong correlation between PPS and OS following treatment with carboplatin and etoposide as first‐line chemotherapy for patients with ED‐SCLC has been previously reported based on individual‐level data.^19^ However, since ICIs have only recently been administered to ED‐SCLC patients, the correlation between PPS and OS in the context of ICI treatment is yet to be elucidated. In addition, the impact of PPS in patients with ED‐SCLC treated with AteCE remains unknown. Thus, there is a need to analyze the correlations between PFS‐OS and PPS‐OS beyond first‐line AteCE for patients with ED‐SCLC using individual‐level data.

This study aimed to retrospectively assess the correlation between both PFS and PPS with OS in patients with ED‐SCLC treated with AteCE. The patients included in our study were a population with limited subsequent treatment choices. We also assessed the clinical factors of patient characteristics for PPS.

## METHODS

### Patients

**FIGURE 1:**
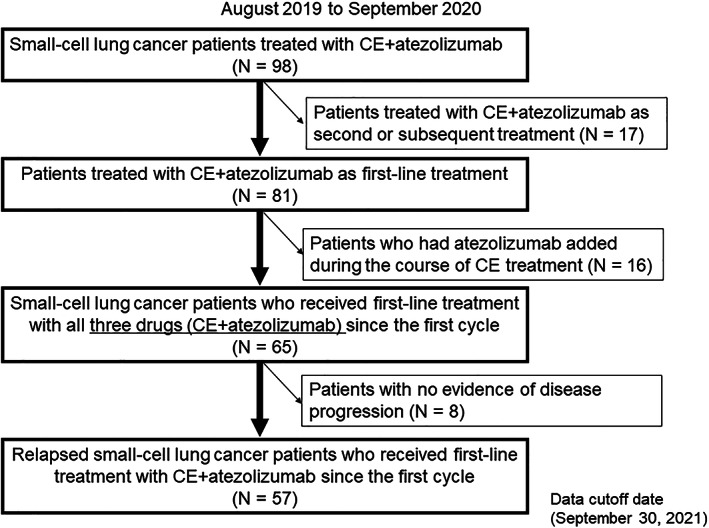
Diagram showing patient selection. Patients treated with atezolizumab plus carboplatin and etoposide between August 2019 and September 2020. CE, carboplatin and etoposide

Between August 2019 and September 2020, 57 patients with ED‐SCLC were retrospectively enrolled in our study at nine Japanese institutions. Eligibility criteria were as follows: cytologic or histologic SCLC diagnosis, inoperable stage III/IV or postoperative recurrence disease at first‐line therapy, first‐line treatment with AteCE, and clinical assessment of disease progression since first‐line AteCE chemotherapy initiation. Figure 1 shows how the patients were selected. Before receiving therapy, all patients underwent systematic evaluation and standardized staging procedures. The clinical stage was assigned based on the results of physical examination, chest radiography, thoracic and abdominal computed tomography (CT), brain magnetic resonance imaging or CT, and bone scintigraphy or ^18^F‐fluorodeoxyglucose positron emission tomography to assess the tumor‐node‐metastasis (TNM) stage. Clinical stage III/IV SCLC was evaluated per the Union for International Cancer Control TNM classification, eighth edition. Data were extracted from the medical charts of eligible patients. The data of the patients who were treated with AteCE were collected as previously described.^20^ This study protocol was approved by the Institutional Review Board of International Medical Center, Saitama Medical University (no. 2021–113). All procedures complied with the ethical standards of the institutional and/or national research committee and with the 1964 Declaration of Helsinki and its subsequent amendments, or comparable ethical standards. Because of the retrospective nature of this study, the informed consent requirement was waived.

### Treatments

All patients had not previously received AteCE combination therapy, and the basic treatment regimen comprised atezolizumab (fixed dose 1200 mg intravenously on day 1 of each cycle), carboplatin (area under the curve 4–5 min mg/ml intravenously on day 1 of each cycle), and etoposide (body surface area 80–100 mg/m^2^ intravenously on days 1–3 of each cycle) for up to four cycles, followed by maintenance atezolizumab administration every 21 days. Granulocyte colony‐stimulating factor was administered at the discretion of the attending physician as neutropenia prophylaxis. Treatment was ended if the disease progressed, if unacceptable adverse events occurred, or if the patient withdrew consent to treatment.

### Assessment of treatment efficacy

Radiographic treatment responses were assessed according to the best overall response and maximum tumor reduction based on RECIST version 1.1.^21^ Treatment responses were evaluated as complete response (CR), partial response (PR), stable disease (SD), progressive disease (PD), or not evaluated. If PD was observed, patients who failed treatment were administered subsequent treatment if they wished, including the continuation of atezolizumab maintenance administration. PFS was calculated as the period from the start of AteCE administration until PD or death due to any cause. PPS was calculated as the period from PD to death for AteCE treatment, or censored cases in which no death event occurred within the observation period were censored on the date of the last visit or follow‐up. OS was calculated as the period from the first day of AteCE administration to death, or censored cases in which no death event occurred within the observation period were censored at the date of the last visit or follow‐up.

### Treatment‐free interval

Since treatment‐free interval (TFI) has been reported to be a predictor of second‐line treatment,^22^, ^23^ analysis was performed by classifying patients according to TFI. TFI was defined as the duration from the date of completion of first‐line chemotherapy to the first recurrence. In many cancer studies, SCLC patients—with a TFI of ≥90 days—who relapsed were classified as those with sensitive relapses. In our study, patients who responded to first‐line anticancer treatment and relapsed ≥90 days beyond cytotoxic drug therapy were classified as having sensitive relapses, while patients who did not respond to first‐line cytotoxic drug treatment or relapsed <90 days since treatment completion were classified as having refractory relapses.

### Statistical analysis

Survival curves were drawn according to the Kaplan–Meier method, and PPS values were compared using the log‐rank test. Spearman's rank correlation analysis and linear regression analysis were used to analyze and evaluate correlations. For univariable and multivariable prognostic assessment of the potential clinical factors for PPS, we applied the Cox proportional hazards model with a stepwise regression procedure. Hazard ratios (HR) and 95% confidence intervals (CI) were estimated. Statistically significant differences were determined using a two‐tailed *p*‐value of <0.05. JMP version 11.0 for Windows (SAS Institute) was used for all statistical analyses in this study.

## RESULTS

### Patient backgrounds and therapeutic effectiveness

**FIGURE 2:**
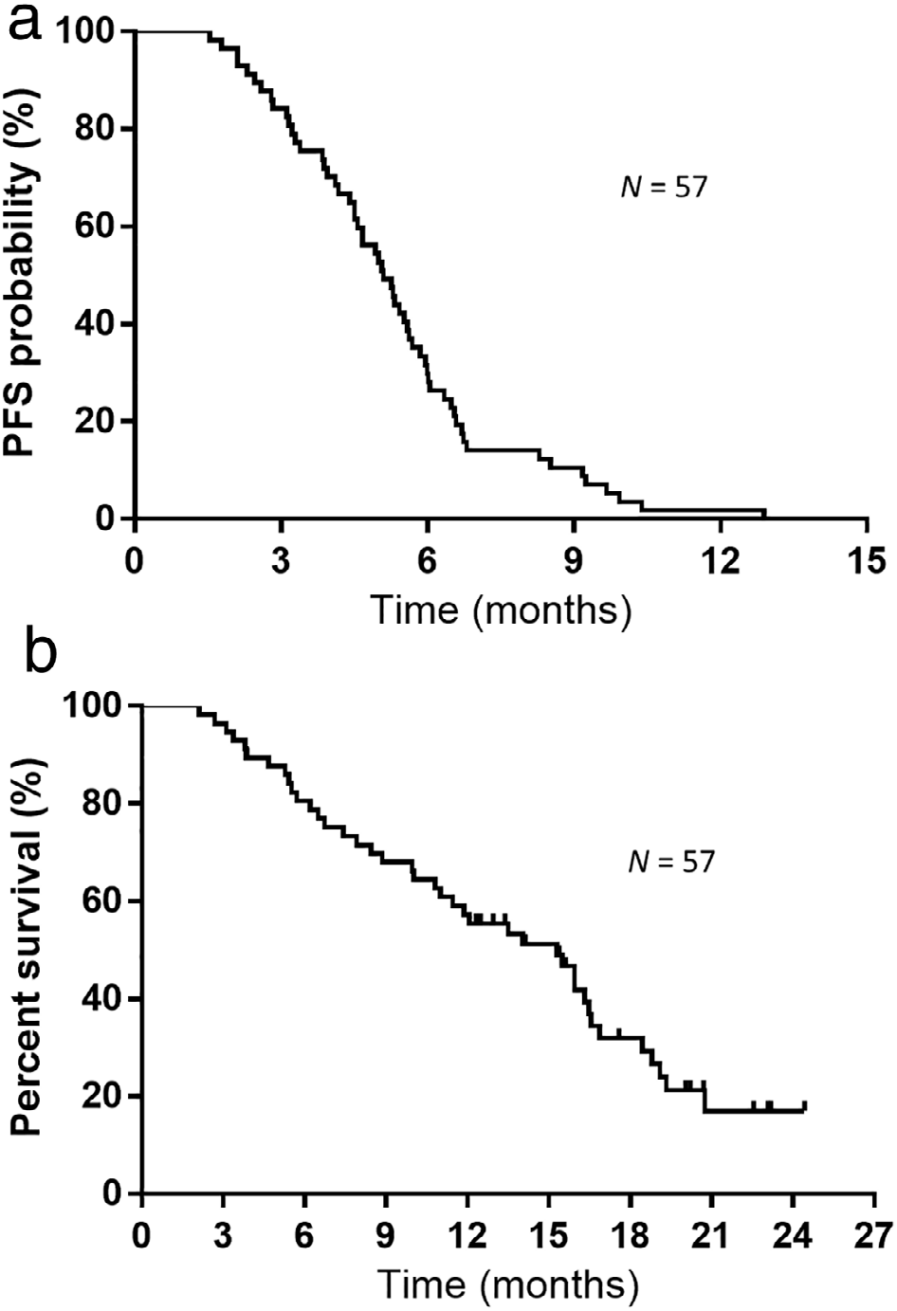
(a) Kaplan–Meier curves of progression‐free survival (PFS). Median progression‐free survival: 5.0 months. (b) Kaplan–Meier curves of overall survival (OS). Median overall survival: 15.2 months

Patient characteristics are shown in Table 1. Among the 57 patients (median age, 70 years; range, 43–86 years) included in our investigation, during a median follow‐up period of 12.9 (range, 2.1–24.4) months, 40 patients died. Regarding treatment response, CR, PR, SD, and PD were achieved in four, 37, 10, and six patients (shown in Table A), respectively. ORR was 71.9% (95% CI: 59.0–81.9), and the disease control rate was 89.4% (95% CI: 78.5–95.4). Regarding survival benefit, the median PFS and OS were 5.0 and 15.2 months, respectively (Figure 2a,b). Among the 57 patients who developed relapse beyond AteCE combination therapy, 14 did not receive further subsequent anticancer drug treatment. Of the 57 patients, the median number of subsequent chemotherapeutic treatments administered following PD after the first‐line treatment was one (range, 0–6 regimens). The chemotherapeutic treatments administered in patients who developed relapse following AteCE combination therapy are listed in Table 2. Amrubicin monotherapy was most frequently used for second‐line treatment, and topotecan monotherapy was most frequently used for third‐line treatment. One patient was still receiving four cycles of maintenance atezolizumab at the data cutoff for beyond PD. The patient was allowed beyond PD continuation of atezolizumab because of slow progression.

**TABLE 1:** Patient characteristics

Characteristic	*N* = 57
Sex	
Male/female	48/9
Age (years)	
Median	70
Range	43–86
ECOG‐PS	
0/1/2/3/4	11/39/5/2/0
Smoking status	
Yes/No	54/3
Histology	
Small cell carcinoma/combined small cell carcinoma	56/1
Disease stage	
III/IV/postoperative recurrence	1/54/2
History of postoperative adjuvant chemotherapy	
Yes/No	1/56
Intracranial metastases at initial treatment	
Yes/No	16/41
Liver metastases at initial treatment	
Yes/No	13/44
Bone metastases at initial treatment	
Yes/No	23/34
Number of cycles of carboplatin + etoposide + atezolizumab administered	
Median	4
Range	1–4
Number of cycles of atezolizumab maintenance therapy administered	
Median	2
Range	0–12
Starting dose	
CBDCA (AUC 5) + etoposide (100 mg/m^2^)	43
CBDCA (AUC 5) + etoposide (80–99 mg/m^2^)	6
CBDCA (AUC 5) + etoposide (<80 mg/m^2^)	1
CBDCA (AUC 4) + etoposide (80 mg/m^2^)	7
With or without G‐CSF prophylaxis	
Yes/No	26/31
Prior radiotherapy	
Yes/No	3/54
Type of relapse	
Sensitive/refractory	21/36
Reason for discontinuation of carboplatin + etoposide + atezolizumab administration^a^	
Progressive disease	7
Adverse events	2
Patient's refusal	1
Immune‐related adverse events	
Yes/No	10/47
Steroid treatment for adverse events^b^	
Yes/No	4/53
Median follow‐up period (months) (range)	12.9 (2.1–24.4)

Abbreviations: AUC, area under the curve; CBDCA, carboplatin; ECOG‐PS, Eastern Cooperative Oncology Group‐Performance Status; G‐CSF, granulocyte colony‐stimulating factor.

^a^ Excluding atezolizumab maintenance therapy.

^b^ Excluding topical agents.

**TABLE 2:** Chemotherapy regimens administered following disease progression after first‐line chemotherapy

	Second‐line	Third‐line	Fourth‐line	≥Fifth‐line	Total^a^
Amrubicin	34	3	1	0	38
Topotecan	1	11	2	1	15
Irinotecan	1	3	3	1	8
CBDCA + etoposide	4	1	1	1	7
CDDP + irinotecan	2	0	0	1	3
CBDCA + paclitaxel	0	0	1	2	3
Others	0	1	0	1	2
Beyond atezolizumab	1	‐	‐	‐	
Best supportive care	14	‐	‐	‐	

Abbreviation: CBDCA, carboplatin; CDDP, cisplatin.

^a^ Total number of patients.

### Correlations between PFS‐OS and PPS‐OS

**FIGURE 3:**
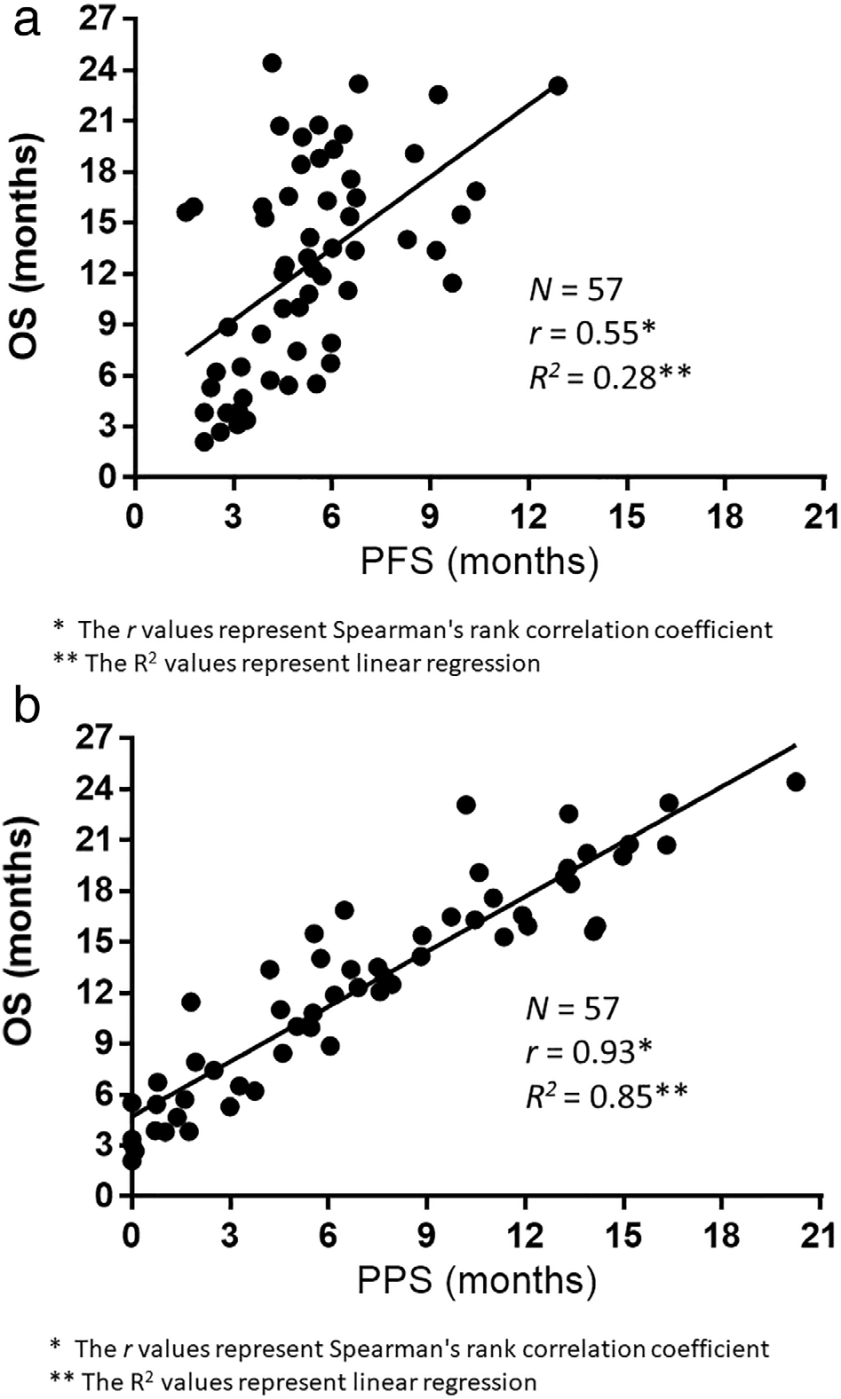
(a) Correlation between overall survival (OS) and progression‐free survival (PFS). (b) Correlation between overall survival (OS) and post‐progression survival (PPS)

**FIGURE 4:**
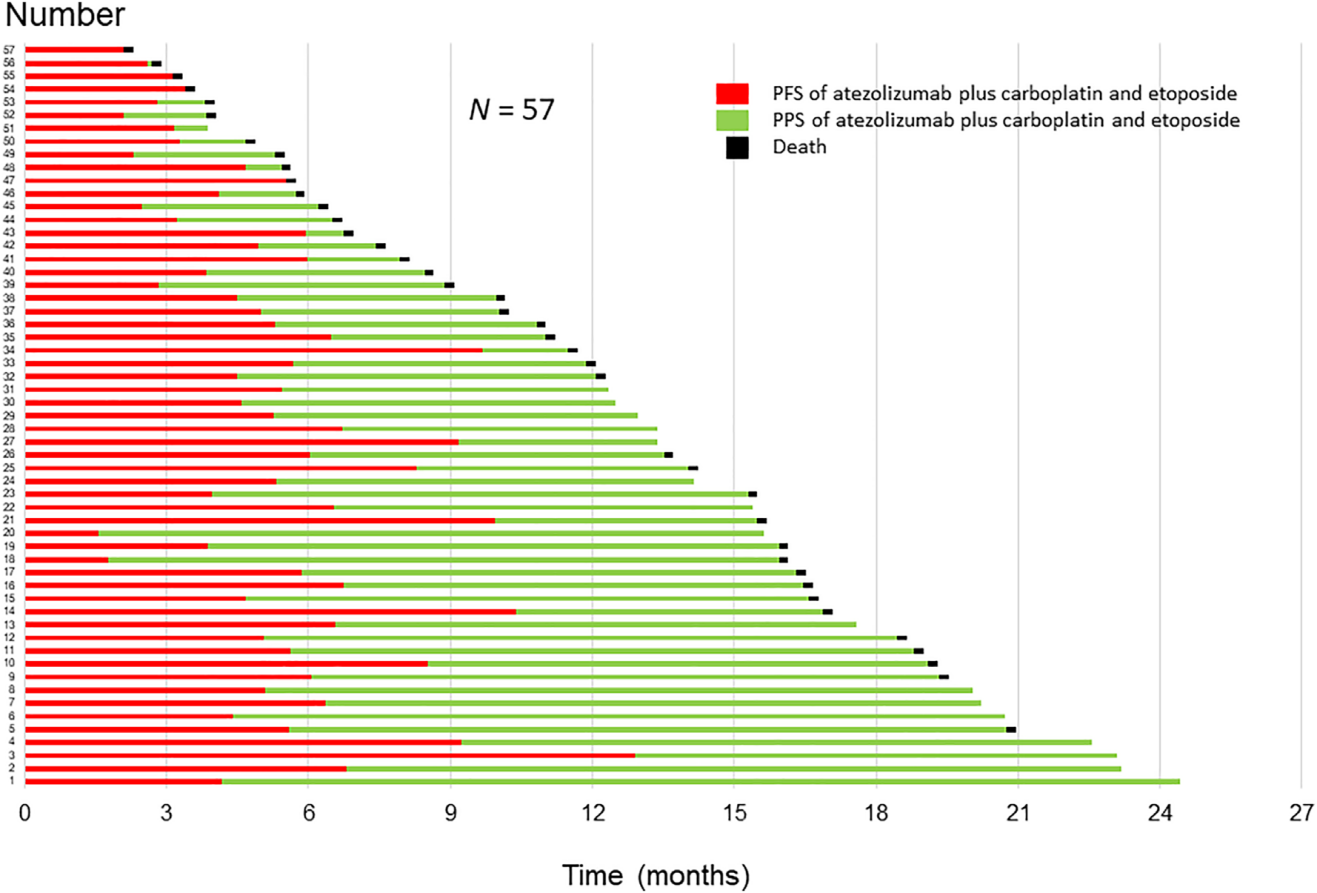
Progression‐free survival (PFS) and post‐progression survival (PPS) in the entire population

The correlations between PFS‐OS and PPS‐OS are demonstrated in Figure 3a,b, respectively. Specifically, Spearman's rank correlation coefficient and linear regression revealed that PPS was highly correlated with OS (*r* = 0.93, *p* < 0.05, *R* ^*2*^ = 0.85), whereas PFS was only moderately correlated with OS (*r* = 0.55, *p* < 0.05, *R* ^*2*^ = 0.28). On the other hand, as shown in Figure S1, Spearman's rank correlation coefficient and linear regression revealed a low correlation between PFS and PPS (*r* = 0.27, *p* = 0.03, *R* ^*2*^ = 0.03). The duration of PFS and PPS in the entire population is shown in a swimmer plot graph (Figure 4).

### Clinical factors influencing PPS

**FIGURE 5:**
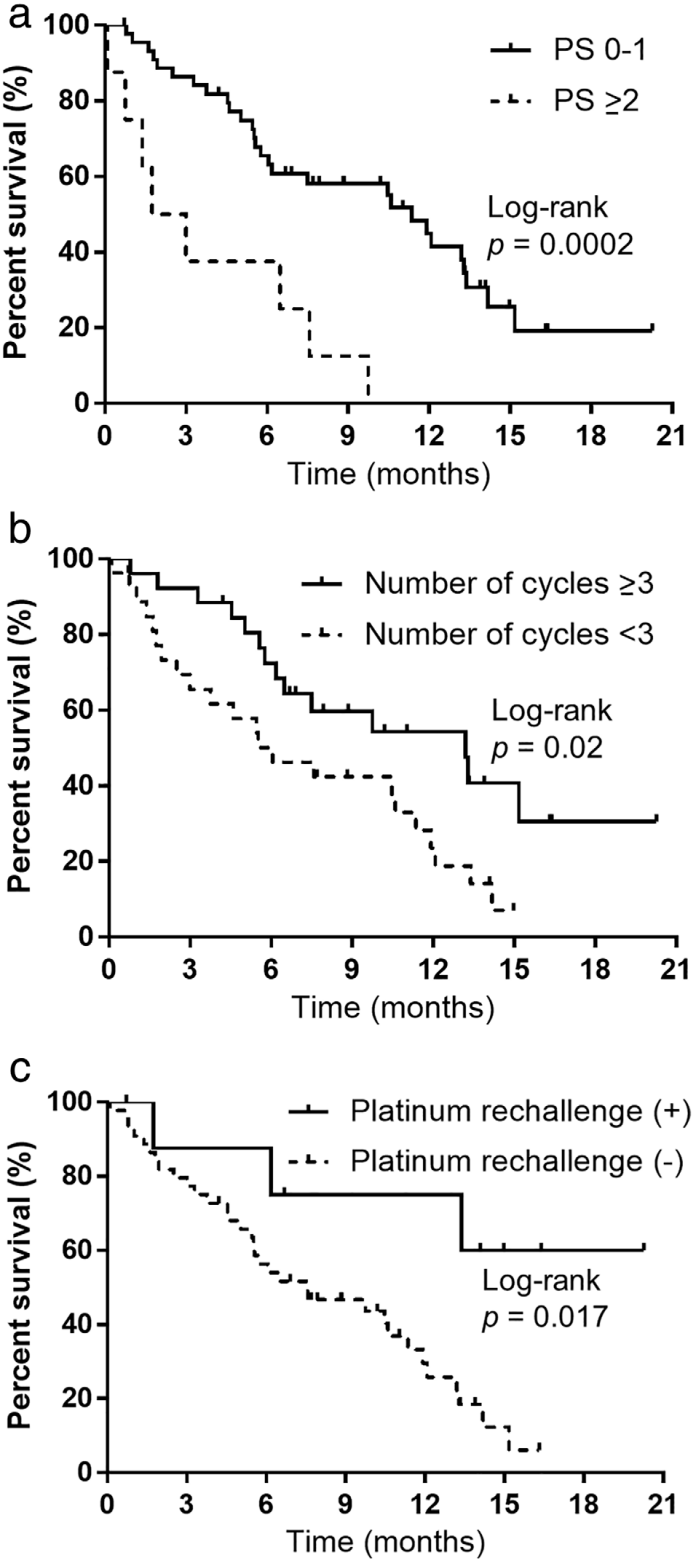
(a) Kaplan–Meier curves of post‐progression survival (PPS), according to the performance status (PS) at relapse. PS 0–1, median = 11.3 months; PS ≥2, median = 2.3 months. (b) Kaplan–Meier curves of post‐progression survival (PPS), according to number of cycles of atezolizumab maintenance therapy. Number of cycles of atezolizumab maintenance therapy ≥3, median = 13.1 months; number of cycles of atezolizumab maintenance therapy <3, median = 5.4 months. (c) Kaplan–Meier curves of post‐progression survival (PPS), according to administration of platinum rechallenge chemotherapy. Platinum rechallenge chemotherapy, median = not reached; no platinum rechallenge chemotherapy, median = 6.0 months

In our analysis, PPS was significantly and strongly correlated with OS. We evaluated correlations with various clinical factors to explore the factors affecting PPS. As shown in Table 3, according to univariate analysis, performance status (PS) at relapse, number of atezolizumab maintenance therapy cycles, administration of platinum rechallenge chemotherapy, administration of amrubicin monotherapy, administration of topotecan monotherapy, and administration of irinotecan monotherapy were all positively correlated with PPS (*p* < 0.05). Furthermore, according to multivariate analysis of PPS (Table 3), ECOG‐PS at relapse, number of cycles of atezolizumab maintenance therapy, and administration of platinum rechallenge chemotherapy were statistically correlated with PPS (*p* < 0.05). We verified that PPS was significantly related to ECOG‐PS at relapse, number of atezolizumab maintenance therapy cycles, and administration of platinum rechallenge by the log‐rank test (*p* < 0.05; Figure 5a–c). Based on the PS at relapse, patients with PS 0–1 showed a median PPS of 11.3 months, which was longer than that of those with a poor PS (PS ≥2; PPS, 2.3 months) (log‐rank test, *p* = 0.0002; Figure 5a). Patients with ≥3 cycles of atezolizumab maintenance therapy had a PPS of 13.1 months, which was longer than that of patients with cycles with shorter administration times; patients with cycles of atezolizumab maintenance therapy administered over a period <3 months had a PPS of 5.4 months (log‐rank test, *p* = 0.02; Figure 5b). Although the median PPS for patients receiving platinum rechallenge chemotherapy was not reached because of the lack of mortality events in more than half of the patients, it is clearly longer than the median PPS of 6.0 months for patients who did not receive rechallenge chemotherapy (log‐rank test, *p* = 0.017; Figure 5c). These results are consistent with those of the adjusted Cox proportional hazards models (Table 3).

**TABLE 3:** Univariate and multivariate Cox regression analysis of patient characteristics for post‐progression survival

		Post‐progression survival					
	Median PPS	Univariate analysis			Multivariate analysis		
Factors	(months)	HR	95% CI	*p*‐value	HR	95% CI	*p*‐value
Sex							
Male/female	7.5/6.4	0.89	0.39–2.37	0.80			
Age at relapse							
<75/≥75	6.1/10.4	1.24	0.61–2.80	0.55			
PS at relapse							
0–1/≥2	11.3/2.3	0.20	0.09–0.44	**0.0002** *	0.28	0.11–0.71	**0.0079** *
Response of atezolizumab plus carboplatin and etoposide							
PR/non‐PR	7.5/3.5	0.55	0.28–1.10	0.09			
Type of relapse							
Sensitive/refractory	11.3/5.5	0.61	0.30–1.16	0.14			
Number of cycles of atezolizumab maintenance therapy							
<3/≥3	5.4/13.1	2.24	1.18–4.44	**0.0134** *	2.35	1.22–4.71	**0.0103** *
Intracranial metastases							
Yes/No	9.7/5.7	0.87	0.42–1.68	0.70			
Liver metastases							
Yes/No	5.4/9.7	1.46	0.65–2.98	0.33			
Bone metastases							
Yes/No	7.4/6.4	0.82	0.42–1.55	0.55			
Immune‐related adverse events of atezolizumab plus carboplatin and etoposide							
Yes/No	13.3/6.4	0.51	0.17–1.20	0.13			
Administration of platinum rechallenge							
Yes/No	NR/6.0	0.24	0.05–0.68	**0.0048** *	0.26	0.06–0.79	**0.0152** *
Administration of amrubicin							
Yes/No	11.3/2.4	0.42	0.22–0.81	**0.0115** *	0.93	0.39–2.24	0.86
Administration of topotecan							
Yes/No	13.1/5.5	0.41	0.18–0.84	**0.0141** *	0.74	0.29–1.76	0.50
Administration of irinotecan							
Yes/No	13.3/6.0	0.40	0.13–0.95	**0.0387** *	0.60	0.19–1.56	0.31

Abbreviations: CI, confidence interval; HR, hazard ratio; PPS, post‐progression survival; PR, partial response; NR, not reported; PS, performance status.

* Statistically significant *p* < 0.05.

## DISCUSSION

In patients with ED‐SCLC, AteCE was administered as first‐line treatment, and the associations between OS‐PFS and OS‐PPS were examined at the individual patient level. Spearman's rank correlation coefficient and linear regression analysis demonstrated that PPS was strongly correlated with OS, while PFS was moderately correlated with OS. In addition, PS at relapse (0–1/≥2), the number of cycles of atezolizumab maintenance therapy (<3/≥3), and the platinum rechallenge chemotherapy independently influenced PPS. This is the first analysis of PPS and its associated influencing factors after first‐line chemotherapy with ICI plus cytotoxic agents in individual‐level ED‐SCLC patients.

Biostatisticians previously described various methods for assessing the validity of alternative endpoints.^24^, ^25^ In ED‐SCLC patients, PFS has been reported to correlate with OS and may be a surrogate endpoint for survival efficacy,^26^, ^27^ but its adequacy remains controversial. One report discusses PPS (= OS minus PFS) in a hypothetical clinical trial setting, assuming that treatment affects PFS but not PPS.^11^ Several studies found that PPS following first‐line chemotherapy for advanced NSCLC at the clinical trial level is strongly correlated with OS^14^, ^15^ similar studies examined the impact of PPS in ED‐SCLC and advanced NSCLC based on individual patient‐level analysis of the strong correlation between OS and PPS.^16^, ^17^, ^18^, ^19^, ^28^, ^29^

In contrast to the previous reports,^26^, ^27^ we found that PFS does not necessarily reflect OS in patients with ED‐SCLC treated with AteCE, but rather that PPS strongly influences OS. The results show that PFS is shorter than PPS, PPS influences OS intimately, and the relationship is linear. The close association of PPS with OS means that the PFS associated with first‐line AteCE therapy, an important component of OS, does not necessarily have a strong impact on OS; prolonged PPS leads to dilution of the significance of PFS of first‐line therapy on overall OS. Undoubtedly, for cancers with poor prognoses, for example, ED‐SCLC, OS should be adopted as the primary efficacy endpoint for any line of treatment, whether first‐, second‐, or subsequent line treatment. Analysis of PFS and PPS of ED‐SCLC and comparison of the relationship between PFS‐OS and PPS‐OS shows that, unlike in other solid tumors for which long‐lived and effective treatments exist, the important clinical significance of focusing on prolonging first‐line PFS in ED‐SCLC in the study design may not be absolutely high. Therefore, clinical trials for aggressive diseases, for example, ED‐SCLC, which include patients who are expected to have a short PFS upon first‐line treatment, should focus on and control factors that affect PPS. Additionally, PPS is longer than PFS, and PPS needs death events, which makes PPS less useful for prognostication in clinical settings. However, PPS has clinical significance in that subsequent treatment after disease progression following front‐line treatment may have a significant impact on OS and control of subsequent treatment may lead to improved OS.

A PPS‐related analysis of individual ED‐SCLC patients treated with first‐line cisplatin and irinotecan treatment reported that a longer PPS was correlated with tumor response to second‐line chemotherapy and the number of chemotherapeutic regimens administered following PD after first‐line treatment.^28^ An analysis of individual ED‐SCLC patients treated with carboplatin and etoposide treatment as first‐line chemotherapy reported that a longer PPS was correlated with both the sensitive relapse and number of chemotherapeutic regimens administered after PD following first‐line treatment.^19^ Currently, the clinical factors affecting PPS in patients with ED‐SCLC receiving ICI plus platinum and etoposide as first‐line treatment are not clear. Thus, we identified clinical factors affecting PPS at the individual level regarding ED‐SCLC patients treated with AteCE combination therapy. Our analysis found that the PS at relapse (0–1/≥2), number of atezolizumab maintenance therapy cycles (<3/≥3), and the platinum rechallenge chemotherapy were highly correlated with PPS in ED‐SCLC patients treated with AteCE. We additionally analyzed the correlations of these factors with the log‐rank test. The results indicate that good PS at relapse is associated with prolonged PPS after PD following first‐line AteCE treatment. Furthermore, there is a high possibility that anticancer drug therapy after first‐line treatment relapse can continue, and it is possible to extend PPS, which, in turn, may contribute to longer OS. The results of the current analysis confirm that ECOG‐PS is a strong prognostic factor, as previously reported,^30^ suggesting that our study patients reflect the general patient cohort. Our analysis revealed that the commonly reported relapse pattern, sensitive or refractory relapse, is not an independent prognostic factor for PPS. However, patients treated with more cycles of atezolizumab maintenance therapy (≥3) have longer PPS. With regard to the results of the IMpower133 trial,^9^ AteCE treatment is associated with a longer duration of durable response to the additional effect of atezolizumab, which may inevitably lead to a longer PPS since more cycles of atezolizumab maintenance therapy are required. Before the introduction of ICIs, patients with SCLC who responded to initial chemotherapy and had a long interval period between the end of initial therapy and relapse (usually 60–90 days or more) were often classified by relapse timing as “sensitive relapse” and those with a shorter interval as “refractory relapse.” Patients with sensitive relapse had better efficacy to cytotoxic drug treatment at relapse and had longer survival.^31^, ^32^ However, the criteria for sensitive or refractory relapse after ICI treatment may need to be re‐examined per the situation in the current ICI era. In the future, the number of ICI administrations may replace the relapse pattern in determining treatment response. Furthermore, platinum rechallenge chemotherapy results in the extension of PPS. A phase III study (GFPC01‐13) comparing oral topotecan alone with carboplatin and etoposide (platinum rechallenge chemotherapy) in patients with sensitive relapse following treatment with platinum and etoposide combination chemotherapy reported that the primary endpoint, PFS, was significantly longer in the carboplatin and etoposide group (median: 4.7 vs. 2.7 months, HR: 0.57).^33^ Although the study did not include ICIs as the first‐line platinum‐based combination chemotherapy, our results indicate that platinum rechallenge chemotherapy is an independent prognostic factor for PPS after AteCE, and could be a treatment option. In the study of patients with ED‐SCLC treated with first‐line carboplatin and etoposide combination chemotherapy as previously described,^19^ a longer PPS was correlated with the pattern of relapse, sensitive or refractory relapse, and the number of chemotherapeutic regimens administered following PD after the first‐line treatment. However, the type of relapse was not associated with AteCE treatment, and the number of cycles of atezolizumab maintenance therapy was identified as a prognostic value of clinical factors for PPS. The number of chemotherapeutic regimens administered following PD was not included as a factor in our analysis, but the number of atezolizumab maintenance therapy cycles and subsequent therapy regimens was analyzed; platinum rechallenge chemotherapy was identified as an independent prognostic factor for PPS. Although the number of regimens administered after disease progression may increase in correlation with a longer PPS, patients with good PS might have been selected for platinum rechallenge chemotherapy, resulting in a greater number of regimens administered after disease progression. A recent review found that the clinical benefit of ICIs is limited to patients with NSCLC who have a favorable PS, which supports this hypothesis.^34^ Considering our results in terms of prognostic factors related to PPS, the patient's ability to withstand a greater number of cycles of atezolizumab maintenance therapy and platinum rechallenge chemotherapy may be associated with a better PS.

Biomarkers reported for ICIs, such as tumor mutation burden and programmed death‐ligand 1 (PD‐L1) protein expression, are not useful for identifying SCLC patients who will benefit from AteCE combination chemotherapy.^35^ Likewise, biomarkers that are practical and actionable for selecting optimal drug therapy in clinical practice are not currently in clinical application. The lack of biomarkers of ICI in SCLC and the limitations of PD‐L1 immunohistochemical analysis indicate the importance and need for research to further evaluate uncharacterized biomarkers of ICI therapy in SCLC and their association with clinical outcomes.

Our study had some limitations. First, it was a retrospective analysis with a relatively small number of eligible patients. Because different physicians recorded tumor responses, it may be more accurate for future analyses if the assessments of disease progression and tumor response rate are recorded by a single attending physician. Although bias may exist, and the inherent limitations of a retrospective study, the findings can still be considered meaningful. Second, therapy with anticancer drugs was at the discretion of the treating physician; hence, treatment may have been reduced, skipped, or delayed. To minimize such bias, all consecutive patients treated at participating institutions were enrolled in the study, and their clinical records were comprehensively examined. Third, the patient information included cases with censored survival data. However, the existence of censored data should not influence our conclusions. If the patient did not die during the follow‐up time, the duration of PFS was unchanged; PPS and OS became longer, and PPS was accordingly even more strongly correlated with OS.

In conclusion, ED‐SCLC patients treated with AteCE chemotherapy as first‐line treatment display a greater influence of PPS on OS than that of PFS on OS. Additionally, atezolizumab maintenance or chemotherapy rechallenge could affect PPS. However, larger‐scale studies in other patient cohorts and clinical settings are necessary to verify our conclusions.

## CONFLICT OF INTEREST

None of the authors have any financial or personal relationships with other people or organizations that could inappropriately influence this work.

## Supporting information

Supporting Information.

**Figure S1** Supporting Information.

## References

[1] Siegel RL , Miller KD , Fuchs HE , Jemal A . Cancer statistics, 2021. CA Cancer J Clin. 2021;71:7–33. 33433946 10.3322/caac.21654

[2] Govindan R , Page N , Morgensztern D , Read W , Tierney R , Vlahiotis A , et al. Changing epidemiology of small‐cell lung cancer in the United States over the last 30 years: analysis of the surveillance, epidemiologic, and end results database. J Clin Oncol. 2006;24:4539–44. 17008692 10.1200/JCO.2005.04.4859

[3] Bernhardt EB , Jalal SI . Small cell lung cancer. Cancer Treat Res. 2016;170:301–22. 27535400 10.1007/978-3-319-40389-2_14

[4] Johnson BE , Jänne PA . Basic treatment considerations using chemotherapy for patients with small cell lung cancer. Hematol Oncol Clin North Am. 2004;18:309–22. 15094173 10.1016/j.hoc.2003.12.008

[5] Demedts IK , Vermaelen KY , van Meerbeeck JP . Treatment of extensive‐stage small cell lung carcinoma: current status and future prospects. Eur Respir J. 2010;35:202–15. 20044461 10.1183/09031936.00105009

[6] Byers LA , Rudin CM . Small cell lung cancer: where do we go from here? Cancer. 2015;121:664–72. 25336398 10.1002/cncr.29098 PMC5497465

[7] Farago AF , Keane FK . Current standards for clinical management of small cell lung cancer. Transl Lung Cancer Res. 2018;7:69–79. 29535913 10.21037/tlcr.2018.01.16 PMC5835595

[8] Schabath MB , Nguyen A , Wilson P , Sommerer KR , Thompson ZJ , Chiappori AA . Temporal trends from 1986 to 2008 in overall survival of small cell lung cancer patients. Lung Cancer. 2014;86:14–21. 25115410 10.1016/j.lungcan.2014.07.014 PMC4171454

[9] Horn L , Mansfield AS , Szczęsna A , Havel L , Krzakowski M , Hochmair MJ , et al. First‐line Atezolizumab plus chemotherapy in extensive‐stage small‐cell lung cancer. N Engl J Med. 2018;379:2220–9. 30280641 10.1056/NEJMoa1809064

[10] Paz‐Ares L , Dvorkin M , Chen Y , Reinmuth N , Hotta K , Trukhin D , et al. Durvalumab plus platinum‐etoposide *versus* platinum‐etoposide in first‐line treatment of extensive‐stage small‐cell lung cancer (Caspian): a randomised, controlled, open‐label, phase 3 trial. Lancet. 2019;394:1929–39. 31590988 10.1016/S0140-6736(19)32222-6

[11] Broglio KR , Berry DA . Detecting an overall survival benefit that is derived from progression‐free survival. J Natl Cancer Inst. 2009;101:1642–9. 19903805 10.1093/jnci/djp369 PMC4137232

[12] Soria JC , Massard C , Le Chevalier T . Should progression‐free survival be the primary measure of efficacy for advanced NSCLC therapy? Ann Oncol. 2010;21:2324–32. 20497965 10.1093/annonc/mdq204

[13] Reck M , von Pawel J , Zatloukal P , Ramlau R , Gorbounova V , Hirsh V , et al. Phase III trial of cisplatin plus gemcitabine with either placebo or bevacizumab as first‐line therapy for nonsquamous non‐small‐cell lung cancer: AVAil. J Clin Oncol. 2009;27:1227–34. 19188680 10.1200/JCO.2007.14.5466

[14] Hotta K , Kiura K , Fujiwara Y , Takigawa N , Hisamoto A , Ichihara E , et al. Role of survival post‐progression in phase III trials of systemic chemotherapy in advanced non‐small‐cell lung cancer: a systematic review. PLoS One. 2011;6:e26646. 22114662 10.1371/journal.pone.0026646 PMC3219633

[15] Hayashi H , Okamoto I , Morita S , Taguri M , Nakagawa K . Postprogression survival for first‐line chemotherapy of patients with advanced non‐small‐cell lung cancer. Ann Oncol. 2012;23:1537–41. 22039091 10.1093/annonc/mdr487

[16] Imai H , Takahashi T , Mori K , Ono A , Akamatsu H , Shukuya T , et al. Individual‐level data on the relationships of progression‐free survival, post‐progression survival, and tumor response with overall survival in patients with advanced non‐squamous non‐small cell lung cancer. Neoplasma. 2014;61:233–40. 24299320 10.4149/neo_2014_030

[17] Imai H , Yamada Y , Sugiyama T , Minemura H , Kaira K , Kanazawa K , et al. Clinical impact of post‐progression survival on overall survival in elderly patients with non‐small‐cell lung cancer harboring sensitive EGFR mutations treated with first‐line EGFR tyrosine kinase inhibitors. Chemotherapy. 2018;63:181–9. 30107372 10.1159/000490949

[18] Imai H , Kishikawa T , Minemura H , Yamada Y , Ibe T , Mori K , et al. Post‐progression survival influences overall survival among patients with advanced non‐small cell lung cancer undergoing first‐line Pembrolizumab monotherapy. Oncology. 2021;99:562–70. 34237736 10.1159/000516745

[19] Imai H , Mori K , Watase N , Fujimoto S , Kaira K , Yamada M , et al. Clinical significance of the relationship between progression‐free survival or post‐progression survival and overall survival in patients with extensive disease‐small‐cell lung cancer treated with carboplatin plus etoposide. Can Respir J. 2016;2016:5405810–8. 27445549 10.1155/2016/5405810 PMC4942672

[20] Shiono A , Imai H , Wasamoto S , Tsuda T , Nagai Y , Minemura H , et al. Real‐world data of atezolizumab plus carboplatin and etoposide in elderly patients with extensive‐disease small‐cell lung cancer. Cancer Med. 2022. 10.1002/cam4.4938 PMC9844637 35699088

[21] Eisenhauer EA , Therasse P , Bogaerts J , Schwartz LH , Sargent D , Ford R , et al. New response evaluation criteria in solid tumours: revised RECIST guideline (version 1.1). Eur J Cancer. 2009;45:228–47. 19097774 10.1016/j.ejca.2008.10.026

[22] Giaccone G , Donadio M , Bonardi G , Testore F , Calciati A . Teniposide in the treatment of small‐cell lung cancer: the influence of prior chemotherapy. J Clin Oncol. 1988;6:1264–70. 2842464 10.1200/JCO.1988.6.8.1264

[23] Ebi N , Kubota K , Nishiwaki Y , et al. Second‐line chemotherapy for relapsed small cell lung cancer. Jpn J Clin Oncol. 1997;27:166–9. 9255271 10.1093/jjco/27.3.166

[24] Weir CJ , Walley RJ . Statistical evaluation of biomarkers as surrogate endpoints: a literature review. Stat Med. 2006;25:183–203. 16252272 10.1002/sim.2319

[25] Fleischer F , Gaschler‐Markefski B , Bluhmki E . A statistical model for the dependence between progression‐free survival and overall survival. Stat Med. 2009;28:2669–86. 19579225 10.1002/sim.3637

[26] Foster NR , Qi Y , Shi Q , Krook JE , Kugler JW , Jett JR , et al. Tumor response and progression‐free survival as potential surrogate endpoints for overall survival in extensive stage small‐cell lung cancer: findings on the basis of north central cancer treatment group trials. Cancer. 2011;117:1262–71. 20960500 10.1002/cncr.25526 PMC3025267

[27] Foster NR , Renfro LA , Schild SE , Redman MW , Wang XF , Dahlberg SE , et al. Multitrial evaluation of progression‐free survival as a surrogate end point for overall survival in first‐line extensive‐stage small‐cell lung cancer. J Thorac Oncol. 2015;10:1099–106. 26134227 10.1097/JTO.0000000000000548 PMC4493926

[28] Imai H , Mori K , Wakuda K , Ono A , Akamatsu H , Shukuya T , et al. Progression‐free survival, post‐progression survival, and tumor response as surrogate markers for overall survival in patients with extensive small cell lung cancer. Ann Thorac Med. 2015;10:61–6. 25593610 10.4103/1817-1737.146885 PMC4286848

[29] Imai H , Kaira K , Minato K . Clinical significance of post‐progression survival in lung cancer. Thorac Cancer. 2017;8:379–86. 28627767 10.1111/1759-7714.12463 PMC5582459

[30] Capewell S , Sudlow MF . Performance and prognosis in patients with lung cancer. The Edinburgh Lung Cancer Group. Thorax. 1990;45:951–6. 2177921 10.1136/thx.45.12.951 PMC462845

[31] Ardizzoni A , Hansen H , Dombernowsky P , Gamucci T , Kaplan S , Postmus P , et al. Topotecan, a new active drug in the second‐line treatment of small‐cell lung cancer: a phase II study in patients with refractory and sensitive disease. The European Organization for Research and Treatment of cancer early clinical studies group and new drug development office, and the lung cancer cooperative group. J Clin Oncol. 1997;15:2090–6. 9164222 10.1200/JCO.1997.15.5.2090

[32] Kim YH , Goto K , Yoh K , Niho S , Ohmatsu H , Kubota K , et al. Performance status and sensitivity to first‐line chemotherapy are significant prognostic factors in patients with recurrent small cell lung cancer receiving second‐line chemotherapy. Cancer. 2008;113:2518–23. 18780323 10.1002/cncr.23871

[33] Baize N , Monnet I , Greillier L , Geier M , Lena H , Janicot H , et al. Carboplatin plus etoposide *versus* topotecan as second‐line treatment for patients with sensitive relapsed small‐cell lung cancer: an open‐label, multicentre, randomised, phase 3 trial. Lancet Oncol. 2020;21:1224–33. 32888454 10.1016/S1470-2045(20)30461-7

[34] Kaira K , Imai H , Mouri A , Yamaguchi O , Kagamu H . Clinical effectiveness of immune checkpoint inhibitors in non‐small‐cell lung cancer with a poor performance status. Medicina. 2021;57:1273. 34833490 10.3390/medicina57111273 PMC8618581

[35] Liu SV , Reck M , Mansfield AS , Mok T , Scherpereel A , Reinmuth N , et al. Updated overall survival and PD‐L1 subgroup analysis of patients with extensive‐stage small‐cell lung cancer treated with Atezolizumab, carboplatin, and etoposide (IMpower133). J Clin Oncol. 2021;39:619–30. 33439693 10.1200/JCO.20.01055 PMC8078320

