# Supplementary material for: Post‐progression survival after atezolizumab plus carboplatin and etoposide as first‐line chemotherapy in small cell lung cancer has a significant impact on overall survival
Source: Thorac Cancer. 2022 Sep 5;13(19):2776–85. doi: 10.1111/1759-7714.14621 (PMC9527159; doi:10.1111/1759-7714.14621)
Supplement: Supplementary file 1 — Supporting Information. [file TCA-13-2776-s001.docx]

Supplementary Material

**Supplementary Table A. Tumor response**

|  | N = 57 |
| --- | --- |
| Response |  |
| CR | 4 |
| PR | 37 |
| SD | 10 |
| PD | 6 |
| NE | 0 |
| Response rate (%) (95% CI) | 71.9 (59.0–81.9) |
| Disease control rate (%) (95% CI) | 89.4 (78.5–95.4) |

CR, complete response; PR, partial response; SD, stable disease; PD, progressive disease; NE, not evaluated; CI, confidence interval
