# Supplementary figures and images for: Post‐progression survival after atezolizumab plus carboplatin and etoposide as first‐line chemotherapy in small cell lung cancer has a significant impact on overall survival
Source: Thorac Cancer. 2022 Sep 5;13(19):2776–85. doi: 10.1111/1759-7714.14621 (PMC9527159; doi:10.1111/1759-7714.14621)

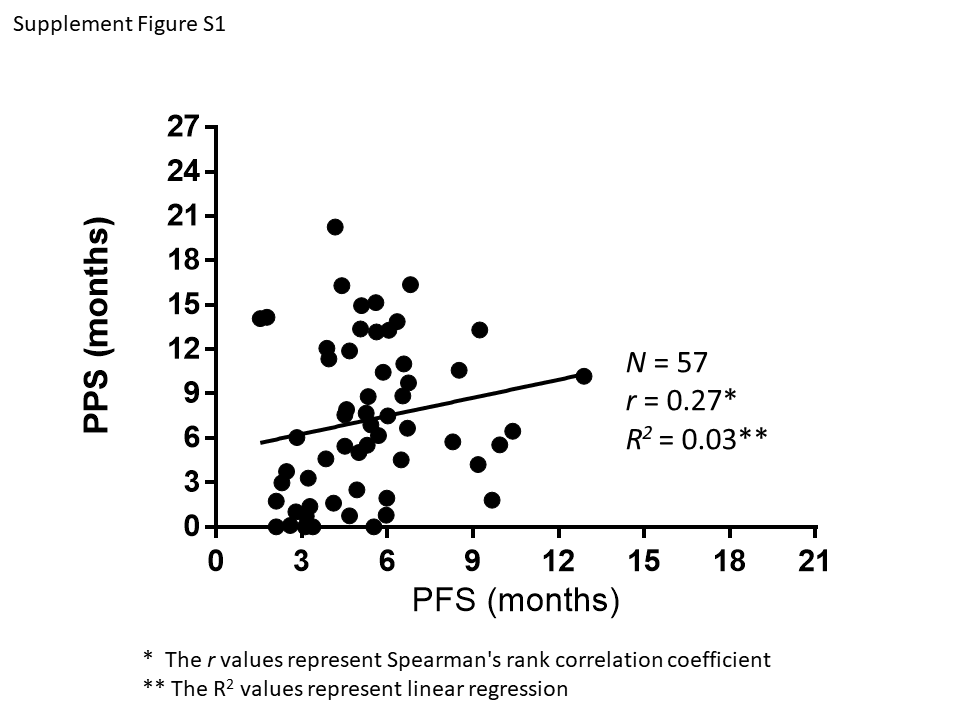

Supplement: Supplementary file 2 — Figure S1 Supporting Information. [file TCA-13-2776-s002.tif]
